# The impact of onabotulinumtoxinA on severe headache days: PREEMPT 56-week pooled analysis

**DOI:** 10.1186/s10194-017-0784-4

**Published:** 2017-08-01

**Authors:** Manjit Matharu, Rashmi Halker, Patricia Pozo-Rosich, Ronald DeGryse, Aubrey Manack Adams, Sheena K. Aurora

**Affiliations:** 1 University College London (UCL) Institute of Neurology and The National Hospital for Neurology and Neurosurgery, Queen Square, London, WC1N3BG UK; 2 Mayo Clinic, Department of Neurology, 5777 East Mayo Blvd, Phoenix, AZ 85054 USA; 3 Headache and Pain Research Group, Institut de Recerca, Universitat Autònoma de Barcelona, Barcelona, Spain; 4 Neurology Department, Vall d’Hebron University Hospital, P.de la Vall d’Hebron, 119-129 08035 Barcelona, Spain; 5 Allergan plc, 2525 Dupont Dr, Irvine, CA USA; 6 Formerly of the Department of Neurology, Stanford University, 300 Pasteur Dr. Room A301 MC 5325, Stanford, CA USA

**Keywords:** Chronic migraine, OnabotulinumtoxinA, Headache severity, Hit-6, PREEMPT

## Abstract

**Background:**

OnabotulinumtoxinA has been shown to reduce headache-days among patients with chronic migraine (CM). The objective of this analysis was to determine whether onabotulinumtoxinA has an impact on headache-day severity in patients with CM among those patients who were deemed non-responders based on reduction in the frequency of headache days alone.

**Methods:**

Data from the Phase 3 REsearch Evaluating Migraine Prophylaxis Therapy (PREEMPT) clinical trial program (a 24-week, 2-treatment cycle, double-blind, randomized, placebo-controlled, parallel-group phase, followed by a 32-week, 3-treatment cycle, open-label phase) were pooled for analysis. Patients kept a daily diary to record headache severity on a 4-point scale (from none to severe), and a 6-domain Headache Impact Test (HIT-6) was used to determine the clinical impact of headaches. Analysis was undertaken to assess whether the subset of patients that were headache-day frequency non-responders at week 24 (patients with <50% reduction in headache-day frequency) experienced a reduction in headache severity whilst receiving onabotulinumtoxinA.

**Results:**

For headache-day frequency non-responders, significant reductions in the number of severe headache days, average daily headache severity, pooled percentage of severe headache days and headache severity score were observed at week 24 for patients who had received onabotulinumtoxinA compared with those who had received placebo. The between-group differences were reduced and non-significant at week 56. Similarly, headache-day frequency non-responders receiving onabotulinumtoxinA were found to have an improvement in the clinical impact of headaches using results from the HIT-6.

**Conclusions:**

These results suggest that even those patients with CM who are deemed non-responders based on analysis of headache frequency alone experience clinically meaningful relief from headache intensity following treatment with onabotulinumtoxinA.

**Electronic supplementary material:**

The online version of this article (doi:10.1186/s10194-017-0784-4) contains supplementary material, which is available to authorized users.

## Background

Chronic migraine (CM; ≥15 headache days per month for ≥3 consecutive months and with ≥8 days/month of migraine-type headaches) [1] is associated with significant personal, societal, and economic burdens [2–5]. Compared with people with episodic migraine (EM; <15 headache days per month), those with CM experience greater headache intensity, increased pain severity and disability, [2] higher rates of comorbid medical conditions, [2, 4] reduced health-related quality of life, [2] greater economic burden, [6] and reduced productivity. [3, 6].

The Phase 3 REsearch Evaluating Migraine Prophylaxis Therapy (PREEMPT) clinical trial program established the safety and efficacy of onabotulinumtoxinA for CM [7–10]. In PREEMPT 1 and 2, patients were randomized to double-blind treatment with onabotulinumtoxinA or placebo (24 weeks), followed by open-label treatment with onabotulinumtoxinA (32 weeks) [7–10]. Treatment with onabotulinumtoxinA resulted in significant improvements in a variety of efficacy endpoints, including the change in frequency of headache days throughout the double-blind treatment period [10]. Nevertheless, anecdotal reports from treating clinicians have indicated that results from these trials do not fully reflect the patient benefits that are observed in clinical practice. Specifically, it has been suggested that onabotulinumtoxinA treatment may have an impact on other clinical characteristics such as headache-day severity.

In this analysis, we assessed the effect of onabotulinumtoxinA on headache-day severity in patients with CM using pooled data from the PREEMPT clinical trials. Our analysis placed a particular focus on the effect on patients who did not experience a clinically meaningful reduction in the frequency of headache days.

## Methods

Study details have been reported previously, [7, 8] and will be only summarized here.

### Study design

Briefly, PREEMPT 1 was conducted at 56 North American sites from January 2006 to July 2008 and PREEMPT 2 was conducted at 50 North American and 16 European sites from February 2006 to August 2008. The studies consisted of a 28-day baseline screening phase, followed by two 12-week treatment cycles over a 24-week randomized, double-blind, placebo-controlled phase (2 treatment cycles), and then a 32-week open-label phase in which all patients received onabotulinumtoxinA (3 treatment cycles).

The PREEMPT clinical trial program was conducted in accordance with the Declaration of Helsinki and Good Clinical Practice guidelines and was approved by an Independent Ethics Committee. All patients provided written informed consent prior to participation in the clinical trial (ClinicalTrials.gov identifiers: NCT00156910 and NCT00168428).

This post-hoc analysis reports pooled results of both PREEMPT 1 and PREEMPT 2 including data from the double-blind and open-label phases of the trials (a total of 56 weeks).

### Study participants

Men and women were eligible for inclusion if they had a history of migraine and met the *International Classification of Headache Disorders* (*2nd edition*; [*ICHD-2*]) migraine diagnostic criteria, with ≥15 headache days per month (headache day was defined as a calendar day with ≥4 continuous hours of headache), of which ≥50% were considered migraine-type headache days. Patients were excluded from the study if they were experiencing continuous headaches, had taken headache prophylaxis in the 4 weeks before enrollment into the study or they had previously been treated with a botulinum toxin.

### Study treatment

Patients were randomized (1:1 in blocks of 4, stratified by frequency of acute pain medication use during the 28-day baseline period) [7, 8] to receive either onabotulinumtoxinA (155 U) or placebo for the first 2 treatment cycles. Therapy was administered via intramuscular injection in fixed dosages at 31 fixed-sites across 7 specific head and neck muscle areas. Up to 40 additional units of onabotulinumtoxinA could be administered according to a “follow the pain” strategy, up to a total dosage of 195 U per treatment cycle administered in up to 39 anatomical sites.

### Assessment of outcome measures

A patient daily telephone diary was kept using an interactive voice response system for the duration of the studies including the 28-day baseline screening period. Headache-day severity was collected on a daily basis via the patient diary and assessed every 4 weeks.

In the PREEMPT trials, pooled safety analyses were undertaken for all patients who received at least one dose of onabotulinumtoxinA or placebo.

#### Headache-day severity

In accordance with guidance for controlled trials, [11] the degree of headache severity was rated on a 4-point scale to indicate severe (3), moderate (2) or mild (1), or headache-free (0). The severity of headache days was determined by the maximum severity across all the headache reports for the day. For headaches that lasted >1 calendar day, the reported level of headache severity was applied to each day that the headache lasted for any given headache report. Diary days that were either without any reported headache or with a reported headache of <4 h continuous duration were defined as headache-free days.

Headache-day severity outcomes assessed the change from the baseline period in the number of days with severe headache (as assessed from patient dairies per 28-day period), pooled number of severe headache days (defined as the sum of severe headache days reported across all patients during the previous 28-day period), and the severity responder analysis. A severity responder was defined as a patient who achieved a ≥ 1-grade improvement in average daily headache severity (ADHS) score across the assessment period (e.g., a reduction in headache severity from severe to moderate).

Headache-day severity outcomes were then assessed among those patients who were considered headache-day frequency “non-responders”. Headache-day frequency non-responders were defined as patients with <50% reduction in headache-day frequency from the 28-day baseline screening period to week 24, as defined by the number of patient diary-reported days per 28-day period with ≥4 continuous hours of headache.

The 6-Item Headache Impact Test (HIT-6), [12, 13] a 6-domain internet-based survey, was used to assess the impact of headaches on the patient. Response options for each of the 6 questions were: never (scored as 6), rarely (8), sometimes (10), very often (11), and always (13), giving a total possible score of between 36 and 78. If ≥50% of the questions were answered, the total score was extrapolated from the mean score across answered questions. If <50% of the questions were answered, the score was set to missing.

Using the HIT-6 outcomes, patient response was analyzed. Patients who had an improvement in headache severity from baseline (≥1-grade improvement in severity from baseline) and an improvement in their HIT-6 score of ≥5 points were considered to be “responders”, as a reduction in HIT-6 scores of ≥5 points from baseline has previously been defined as clinically meaningful [14, 15].

### Statistical analyses

#### Headache-day severity

We assessed the change from baseline in the number of severe headache days. Missing counts were estimated using modified last observation carried forward (mLOCF) techniques. *P*-values for between-treatment comparisons were calculated using covariate analysis of variance (ANCOVA), with baseline values as the covariate. The main effects in the ANCOVA included treatment and medication-overuse strata, where the type III sum of squares was used.

Statistics were calculated for the number of severe headache days pooled across patients for each time period. Any missing time period data for a patient were estimated using mLOCF. Between-treatment comparisons of the percentage of severe headache days were determined by Pearson’s chi-square or Fisher’s exact tests (for this parameter and for others discussed below, if ≥25% of the expected cell counts were <5) for each headache-day severity category.

ADHS scores were the average severity score, as assessed by the patient, across all reported diary days, weighted to account for headache days without severity report and rounded to the nearest whole number. Improvement of ≥1 grade in ADHS (e.g. from severe to moderate) included patients with score reduction of at least 1. Statistics were calculated for the change from the baseline severity score. Missing values were estimated using mLOCF. Between-treatment comparisons of the percentage of patients with ≥1-grade improvement in ADHS were determined by Pearson’s chi-square or Fisher’s exact tests.

#### HIT-6 responder analysis

Similarly, statistics were calculated for baseline and change from baseline for HIT-6 scores to categorize HIT-6 responders, defined as patients with ≥5-point improvement in HIT-6 scores from baseline. For various time periods in the subset of headache-day frequency non-responders, whose headaches had reduced in severity by at least 1 grade by week 24, between-treatment comparisons of HIT-6 responders were determined by Pearson’s chi-square or Fisher’s exact tests.

## Results

### Patient disposition and demographics

**Fig. 1:**
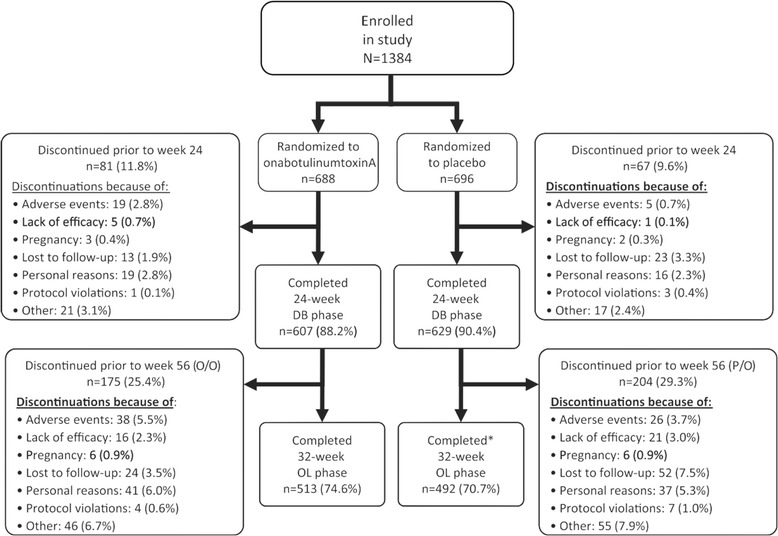
Patient Disposition. Reproduced with permission from Aurora, et al. *Headache* 2011;51:1358–73

A total of 1384 patients received either onabotulinumtoxinA (*n* = 688) or placebo (*n* = 696) in the 24-week double-blind phase before receiving onabotulinumtoxinA in the open label phase. Baseline demographic and clinical characteristics were similar between the two overall treatment groups (Table 1). The mean patient age at baseline was 41 years, and the mean duration since the onset of CM was 19 years. More than 85% of the patients in both groups were female and more than 60% of patients were using prophylactic medications for migraine prior to enrollment into the study. Patient discontinuation across the 56-week study was 25.4% and 29.3% for the onabotulinumtoxinA and placebo study arms, respectively, with no major differences in the reasons for withdrawal from the study (Fig. 1).

**Table 1:** Baseline demographic and clinical characteristics for overall PREEMPT group and the non-responder subgroup^a^

Characteristic	Overall PREEMPT Group			Non-Responder Subgroup		
	O/O (*n* = 688)	P/O (*n* = 696)	*P*-value^b^	O/O (*n* = 285)	P/O (*n* = 360)	*P*-value^b^
Mean (SD) age, y	41.1 (10.4)	41.5 (10.7)	0.58	42.3 (10.4)	42.8 (10.5)	0.56
Age ≥ 40 y, n (%)	395 (57.4)	408 (58.6)	0.65	179 (62.8)	224 (62.2.)	0.94
Sex, n (%)						
Female	603 (87.6)	593 (85.2)	0.19	242 (84.9)	309 (85.8)	0.74
Race/Ethnicity, n (%)			0.60			0.40
White	617 (89.7)	630 (90.5)		257 (90.2)	333 (92.5)	
Black	34 (4.9)	40 (5.7)		15 (5.3)	17 (4.7)	
Hispanic	27 (3.9)	19 (2.7)		9 (3.2)	9 (2.5)	
Other	10 (1.5)	7 (1.0)		4 (1.4)	1 (0.3)	
Mean (SD) age of onset of CM, y	21.2 (11.0)	21.9 (11.9)	0.46	21.3 (11.4)	22.5 (12.1)	0.19
Mean (SD) CM duration, y	19.4 (12.4)	19.0 (12.7)	0.49	20.4 (12.2)	19.7 (12.8)	0.46
Mean (SD) headache days (≥4 h) per 28-day period	19.9 (3.7)	19.8 (3.7)	0.52	20.5 (3.9)	20.4 (4.0)	0.61
Prestudy headache prophylactic use, n (%)	425 (61.8)	454 (65.2)	0.18	197 (69.1)	263 (73.1)	0.29
Acute headache medicine overuse, n (%)	446 (64.8)	460 (66.1)	0.62	195 (68.4)	251 (69.7)	0.73
Mean (SD) HIT-6 score^c^	65.5 (4.1)	65.4 (4.3)	0.64	65.4 (3.8)	65.3 (4.4)	0.84
Patients with severe headache impact						
(HIT-6 total score ≥ 60), %^c^	93.5	92.7	0.57	93.7	92.8	0.75
Mean (SD) MSQ score^d^						
Role restrictive	38.5 (16.6)	38.7 (17.3)	0.97	37.9 (16.7)	38.4 (17.5)	0.67
Role preventive	56.0 (21.2)	56.1 (21.7)	0.83	57.0 (21.9)	56.1 (21.7)	0.62
Emotional functioning	42.1 (24.1)	42.4 (25.0)	0.81	42.7 (23.6)	44.6 (24.7)	0.31

*CM* chronic migraine, *HIT*-6 6-item Headache Impact Test, *MSQ* Migraine-Specific Quality of Life Questionnaire, *O/O* onabotulinumtoxinA in double-blind phase and open-label phase, *P/O* placebo in double-blind phase and onabotulinumtoxinA in open-label phase, *PREEMPT* Phase 3 REsearch Evaluating Migraine Prophylaxis Therapy

^a^Nonresponder group = <50% reduction in headache-day frequency at week 24

^b^ *P*-values are the pairwise *t*-test or the Fisher’s exact test between the O/O vs the P/O groups for each respective population group

^c^HIT-6 scores of 36–49 indicate little or no impact; 50–55, some impact; 56–59, substantial impact; 60–78, severe impact

^d^MSQ v2.1 scores range from 0 (poor) to 100 (good)

Of the 645 patients that were classified as non-responders at week 24 (i.e. had <50% reduction in headache-day frequency), 285 patients had received onabotulinumtoxinA and 360 patients had received placebo. There were no statistically significant differences in the baseline demographic and clinical characteristics between the 2 non-responder groups (Table 1). The non-responder groups were also largely similar to the overall population; although a slightly higher percentage of the non-responders used prophylactic medications at baseline.

### Changes in headache severity in headache-day frequency non-responders

**Fig. 2:**
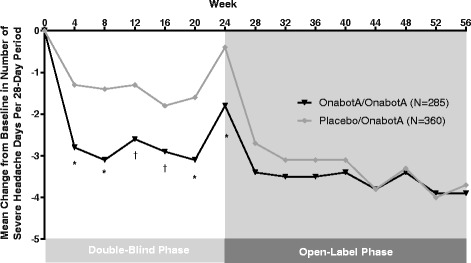
Change from baseline in the number of severe headache days per 28-day period, among nonresponders.*P ≤ 0.001. ^†^ *P* < 0.05

Among those with a less than 50% reduction from baseline in headache-day frequency at week 24 (headache-day frequency non-responders), reduction from baseline in the number of severe headache days per 28-day period was significantly greater when treated with onabotulinumtoxinA compared with placebo throughout the 24-week double-blind period (Fig. 2). These between-group differences decreased and were no longer significant in the open-label phase of the study.

**Fig. 3.**
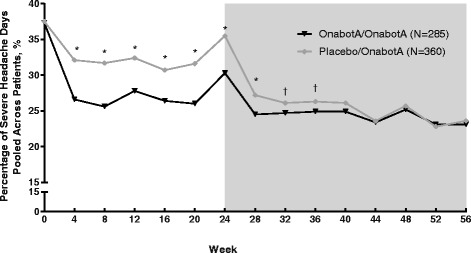
Pooled number of severe headache days, among nonresponders. *P ≤ 0.001. ^†^ *P* < 0.05

In addition, in headache-day frequency non-responders, the percentage of severe headache days pooled across patients was significantly lower throughout the 24-week double-blind period for those treated with onabotulinumtoxinA than those receiving placebo (Fig. 3). These between-group differences decreased during the open-label phase of the trial when both groups of patients received onabotulinumtoxinA and the differences were no longer significant by week 40.

Patients who had at least a 1-grade improvement from baseline in the severity of their headaches based on patient diaries were considered to be severity responders. Among headache-day frequency non-responders, significant reductions in the severity of the headaches occurred more frequently in patients receiving onabotulinumtoxinA than in patients receiving placebo at week 24 (41.1% vs 31.4%; *P* = 0.011; Table 2). Once all patients were receiving onabotulinumtoxinA in the open-label phase of the trial, between-group differences disappeared and were not significant at week 56 (64.6% vs 65.6%; *P* = 0.792).

**Table 2:** Severity and HIT-6 Responder Analysis

*Proportion of severity responders, among headache-day frequency non-responders* ^*a*^			
	OnabotulinumtoxinA/ OnabotulinumtoxinA *n* = 285	Placebo / OnabotulinumtoxinA *n* = 360	*P-*value
Week 24	41.1%	31.4%	0.011
Week 56	64.6%	65.6%	0.792
*Proportion of HIT-6 responders, among severity responders* ^*b*^			
	OnabotulinumtoxinA/ OnabotulinumtoxinA *n* = 246	Placebo / OnabotulinumtoxinA *n* = 161	*P*-value
Week 24	62.2%	43.5%	<0.001
Week 56	74.4%	72.0%	0.601

*HIT-6* 6-item Headache Impact Test

^a^Patients with severity response (≥1-grade improvement from baseline in severity), among patients with <50% reduction from baseline in headache-day frequency at week 24

^b^Patients with a HIT-6 response (≥5-point improvement from baseline), among patients with ≥1-grade reduction from baseline in headache-day severity at week 24

### Analysis based on HIT-6 scores

Separate analysis was undertaken to assess whether HIT-6 scores demonstrated a positive response to treatment (≥5-point increase in HIT-6 scores from baseline, as has previously been deemed to be a positive response to treatment [14, 15]) in patients whose headaches reduced in severity (≥1 grade change in severity). The results were similar to those reported above. At week 24, HIT-6 responder rates were significantly higher for onabotulinumtoxinA than for placebo (62.2% vs 43.5%; *P* < 0.001), and at week 56, the response rates were similar between treatment groups (74.4% vs 72.0%; *P* = 0.601; Table 2).

### Safety

The results of the PREEMPT trials previously published confirmed that onabotulinumtoxinA is safe and well tolerated for the long-term prophylactic treatment of CM [9]. Treatment-related adverse events (TRAEs) were consistent with the known safety profile of onabotulinumtoxinA. In the double- blind phase of the study, neck pain (6.7%), muscular weakness (5.5%), eyelid ptosis (3.3%), injection-site pain (3.2%), headache (2.9%), myalgia (2.6%), musculoskeletal stiffness (2.3%), and musculoskeletal pain (2.2%) were reported by ≥2% of patients receiving onabotulinumtoxinA. TRAE decreased in the open-label phase of the study with neck pain (4.6%), muscular weakness (3.9%), eyelid ptosis (2.5%), muscle tightness (2.2%) and injection site pain (2.0%) the only TRAEs occurring in ≥2% of patients. Serious TRAEs were rare occurring in 1 patient (0.1%) in both the double-blind and open-label phases of the PREEMPT trials.

## Discussion

In an earlier analysis of the PREEMPT data, it was observed that 49% of patients treated with onabotulinumtoxinA demonstrated a ≥ 50% reduction in headache-day frequency after 1 treatment cycle, and an additional 11% who did not respond after the first treatment cycle responded after treatment cycle 2 [16]. In the current analysis, we present data for the effect of onabotulinumtoxinA on headache-day severity among those with CM who did not have a reduction in headache-day frequency after two cycles of treatment (non-responders defined as <50% reduction in headache-day frequency at week 24). During the randomized, placebo-controlled, double-blind phase, patients treated with onabotulinumtoxinA demonstrated a greater reduction from baseline in the number of severe headache days per 28-day period than did those receiving placebo. In addition, compared with the placebo group, there were a lower percentage of patients receiving onabotulinumtoxinA with an ADHS score of severe, a lower percentage of severe headache days pooled across all patients within the onabotulinumtoxinA group, and a higher rate of at least 1-grade improvement in headache severity from baseline (severity responders). Among all severity responders, the proportion of HIT-6 responders (≥5-point improvement from baseline) was greater for the onabotulinumtoxinA group than the placebo group at the end of the double-blind phase.

The headache-day severity endpoints showed a noteworthy peak at week 24 in both onabotulinumtoxinA and placebo treatment groups. The key reason for this non-response peak is the selection criteria, since the population of interest was predefined as those without adequate treatment response in relation to headache-day frequency at week 24. It is interesting that in this group of non-responders, there was some response at other time points both before the arbitrary 24-week non-response point and after that time point.

Open-label results where both groups were receiving onabotulinumtoxinA generally demonstrated lesser between-group differences, with no significant differences between the groups observed by the end of the 56-week study. Conversely, headache-day frequency, as previously reported by Aurora et al., [9] was significantly reduced by onabotulinumtoxinA in the double-blind phase and continued to show between-group differences through to week 56, making a case for early treatment with onabotulinumtoxinA in patients with CM. This observation suggests that treatment with onabotulinumtoxinA produces significant reduction in headache-day severity that may compliment a reduction in headache-day frequency, since onabotulinumtoxinA treatment in the open-label phase was able to eliminate between-group differences in severity, but not frequency. These findings are worthy of further investigation to fully understand the potential therapeutic benefit of early treatment with onabotulinumtoxinA.

Importantly, the current analysis demonstrated reduced headache-day severity at the first post-baseline assessment (week 4) in patients who were headache-day frequency non-responders at week 24, suggesting an important clinical response to treatment not captured by the measure of headache-day frequency reduction. Furthermore, the alignment of the HIT-6 response with the severity response suggests that the reduction in headache-day severity of at least 1 grade was clinically meaningful. Further study is required to determine whether this clinical response translates into a reduction in healthcare resource utilization and broader economic benefits.

The large number of patients with CM included in these double-blind placebo-controlled studies makes these results particularly robust. The use of the voice interactive daily telephone diary encouraged high patient compliance with diary record keeping and captured data without the need for reliance on long-term recall. This would be expected to result in more accurate capture of patient data, as others have shown that current health status can have an impact upon a patient’s recollection of the past [17].

The study is not without its limitations. The lack of an active comparator is a potential limitation. However, the lack of any approved prophylactic treatment for CM makes it difficult to identify an appropriate comparator. The time point to determine headache-day frequency nonresponse was set at week 24, which was an arbitrary time point. The selection of a different time point for the definition of non-response may have resulted in different outcomes. Further clinical trials may be required to understand if a different time point for the determination of headache-day frequency non-response has any clinically meaningful impact on the interpretation of the data presented here.

Similarly, no optimal responder rate for the reduction from baseline in headache-day frequency has been defined for the CM population. Although both 30% and 50% cutoffs for headache measures have been suggested to be clinically meaningful, [11] a 50% cutoff is more commonly used in migraine studies, and provided the rationale for the definition of non-responders for the current analysis. Analysis of the current data using a <30% reduction in headache-day frequency as the definition for non-response produced similar results to those reported here, with those classified as non-responders achieving a significant reduction in headache severity (Additional file 1: Table S1, and Figures S1 and S2). This additional analysis using <30% as the cutoff further strengthens the findings of this analysis.

The International Headache Society has published guidelines for clinical trial assessment of prophylactic treatment for CM, including guidelines on the selection of outcome measures [11]. The primary end-point outcomes recommended by these international experts include headache days with moderate or severe intensity, migraine days, or frequency of migraine episodes. However, despite this international accord on the selection of outcome measures to encourage robust clinical trials, the outcomes may not fully align with the patient’s expectation of therapy. This study demonstrates that frequency day response alone may not be sufficient to determine a clinically meaningful response to therapy, and highlights the need for further work on developing key patient-endorsed outcome measures for the assessment of prophylactic treatment of chronic migraine in particular and headache disorders in general.

## Conclusions

Patients from the PREEMPT clinical trial program who received onabotulinumtoxinA and met our definition for headache-day frequency non-response (<50% reduction in headache-day frequency at week 24) demonstrated significantly reduced headache-day severity (compared with those receiving placebo). Among those who showed reduced headache-day severity, onabotulinumtoxinA also produced greater reduction in headache impact scores. These results suggest that patients with CM experience clinically meaningful relief from headache intensity following treatment with onabotulinumtoxinA, even among those who may not experience a clinically meaningful reduction in the frequency of their headaches.

## Additional file

Additional file 1: Table S1. Percentage of patients with a measurable change in headache severity among patients with <30% reduction in headache-day frequency. Figure S1. Change from baseline in the number of severe headache days per 28-day period, among patients with <30% reduction in headache-day frequency. **P*<0.001, †*P*<0.05. Figure S2. Percentage of severe headache days, pooled across patients with <30% reduction in headache-day frequency. **P*<0.001, †*P*<0.05*. (PDF 35 kb)*

## Abbreviations

ADHS: Average daily headache severity
ANCOVA: Analysis of covariance
CM: Chronic migraine
HIT-6: 6-Item Headache impact test
*ICHD-2*: *International classification of headache disorders* (*2nd Edition)*
mLOCF: Modified last observation carried forward
PREEMPT: Phase 3 REsearch Evaluating Migraine Prophylaxis Therapy
TRAE: Treatment-related adverse event
