# Supplementary material for: The impact of onabotulinumtoxinA on severe headache days: PREEMPT 56-week pooled analysis
Source: J Headache Pain. 2017 Aug 1;18(1):78. doi: 10.1186/s10194-017-0784-4 (PMC5539058; doi:10.1186/s10194-017-0784-4)

Supplementary Table. Percentage of patients with a measurable change in headache severity among patients with <30% reduction in headache-day frequency.

Patients with severity response (≥1-grade improvement from baseline in severity based on patient diary)			
	**OnabotulinumtoxinA / OnabotulinumtoxinA n=194**	**Placebo / OnabotulinumtoxinA n=261**	***P*-value**
**Week 24**	24.2%	13.8%	0.004
**Week 56**	54.1%	57.9%	0.428

Supplementary Figure 1. Change from baseline in the number of severe headache days per 28-day period, among patients with <30% reduction in headache-day frequency.
*P<0.001, †P<0.05.

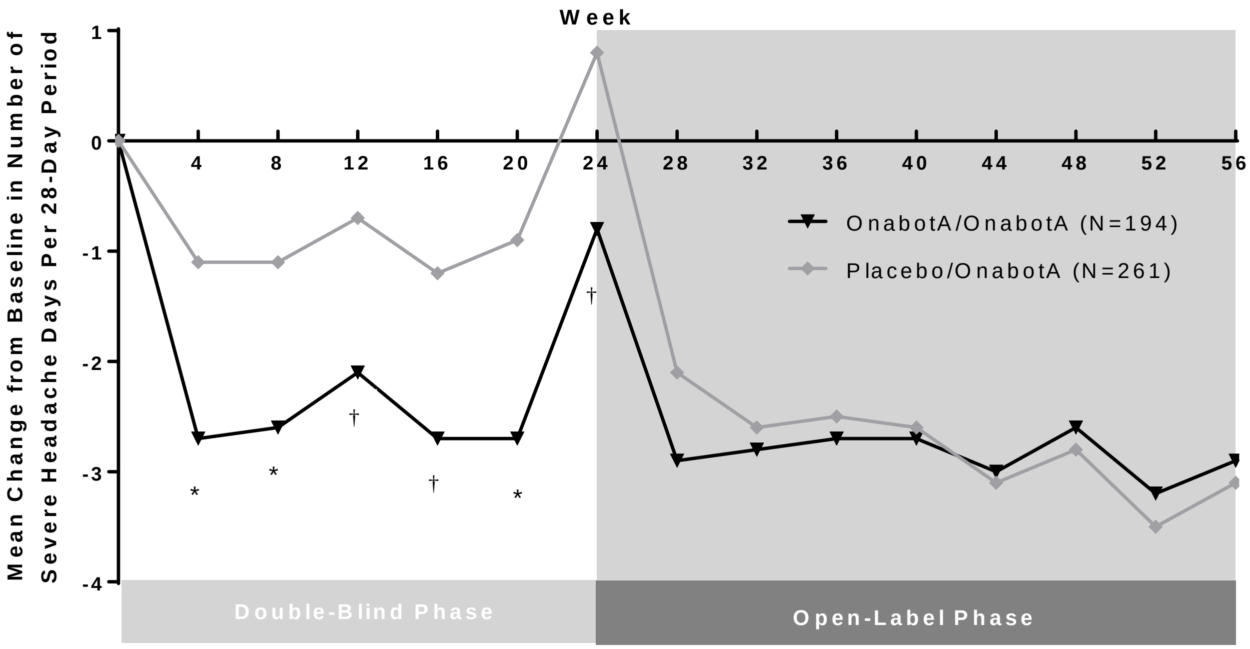

Supplementary Figure 2. Percentage of severe headache days, pooled across patients with <30% reduction in headache-day frequency.
*$P<0.001$, †$P<0.05$.

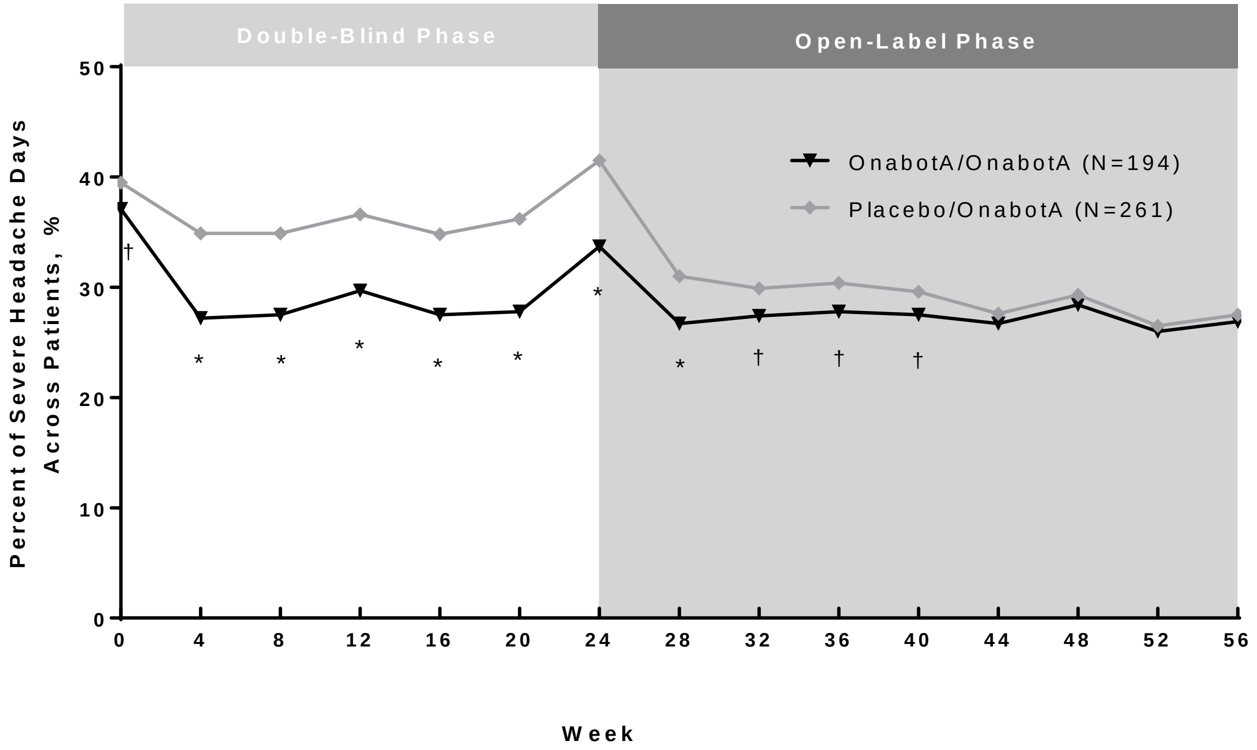

Supplement: Additional file 1: Table S1. — Percentage of patients with a measurable change in headache severity among patients with <30% reduction in headache-day frequency. Figure S1. Change from baseline in the number of severe headache days per 28-day period, among patients with <30% reduction in headache-day frequency. *P<0.001, †P<0.05. Figure S2. Percentage of severe headache days, pooled across patients with <30% reduction in headache-day frequency. *P<0.001, †P<0.05. (PDF 35 kb) [file 10194_2017_784_MOESM1_ESM.pdf]
